# Exploring the Design Space of Glanceable Smartwatch Feedback Displays: Experimental Study

**DOI:** 10.2196/81972

**Published:** 2026-07-23

**Authors:** Yuxuan Li, Mark Newman, Predrag Klasnja

**Affiliations:** 1 School of Information University of Michigan Ann Arbor, MI United States

**Keywords:** glanceable displays, health informatics, mobile health, physical activity, smartwatches

## Abstract

**Background:**

Self-monitoring technologies are commonly used to promote health behavior change, with glanceable displays offering continuous feedback throughout the day. Yet, it is still unclear how various aspects of these glanceable representations affect their interpretability and usability.

**Objective:**

This study aimed to investigate the effects of 3 design factors—stylization, granularity, and salience—on users’ ability to understand glanceable smartwatch-based feedback on daily step goals.

**Methods:**

We conducted an online simulation study to examine how 3 design dimensions—stylization, salience, and granularity—influence the effectiveness of glanceable feedback displays. Stylization and salience were crossed in a 2×2 factorial design, while granularity varied from 1% to 20% progress increments. A total of 202 Amazon Mechanical Turk participants were randomly assigned to 1 of 16 smartwatch display conditions. In each condition, participants viewed feedback on daily step progress and estimated the level of progress shown. We measured estimation error and questionnaire-assessed perceived usability and acceptability. The collected data were analyzed using generalized estimating equations and linear regression.

**Results:**

High stylization reduced accuracy (+4.52 error points; *P*<.001) and negatively affected perceptions across 6 dimensions, including comprehension (*P*=.003), complexity (*P*<.001), and usability (*P*=.001). Granularity had a nonlinear effect: error was lowest around 5%-10%, with sharp increases at 20%. The 10% level also received the most favorable ratings, for example, comprehension (+0.656; *P*=.003). Salience had no effect. Previous smartwatch users were less accurate than never-users (+7.46 points) but rated displays as more useful (*P*=.002) and easier to focus on (*P*<.001). Current users gave similarly positive ratings on attention and usefulness.

**Conclusions:**

These findings could help researchers design effective glanceable smartwatch feedback displays and expand the design space for glanceable feedback.

## Introduction

Self-monitoring is widely used to support behavior change in health-related contexts, and meta-analyses have found self-monitoring to be highly effective for physical activity, diet, and weight loss [1-4]. Increasingly, mobile devices such as smartphones and smartwatches have become a common way of providing self-monitoring interventions [5-8]. One of their key strengths for supporting self-monitoring is that they enable users to see self-monitoring feedback frequently—increasing its salience and, thus, its potential to shape users’ behavior. Mobile devices can increase feedback frequency by leveraging multiple modalities, including inside apps, push notifications, text messages, and, crucially, glanceable displays such as lock screens (phones) and clock faces (smartwatches).

Glanceable displays refer to the designs that present important, noncritical information in an always-visible but nonintrusive form, allowing data to move seamlessly from the periphery of a user’s awareness to the focus of attention and back again [9]. Glanceable displays are a promising approach to supporting self-monitoring, as they enable researchers and intervention designers to create feedback representations that a person can see throughout the day without having to remember to go into an app. By ensuring feedback is always visible, these displays allow users to maintain awareness of their health metrics throughout the day without the burden of needing to navigate to an app or manage incessant push notifications [10-12].

As the smartwatch market has matured, health and fitness have become key domains supported by these devices, and glanceable displays are a central medium through which smartwatches support self-monitoring of health-related metrics. For instance, the Apple Watch includes a complication, which is an element of the clock face that displays information other than the time, for the Activity app, shown by default on most built-in clock faces, which displays feedback on minutes of activity, calories burned, and the number of hours of nonsedentary time. Similarly, most default Fitbit clock faces display step count and heart rate. The information provided in such feedback displays is visible every time the user looks at the watch, providing ongoing awareness about one’s level of activity. Moreover, Apple Watch, Fitbit, Garmin, and Wear OS platforms all support some level of clock face customization, enabling a range of feedback—as well as forms of that feedback—to be incorporated into the clock face. Given how frequently watch wearers glance at their watches each day, for a growing number of smartwatch users, feedback shown on their watches represents a powerful way to benefit from self-monitoring without the burden of receiving incessant notifications.

However, little is known about what makes a glanceable display effective, and, specifically, what design dimensions influence the understandability, usability, and behavioral impact of such displays. Prior research has demonstrated the potential of glanceable displays but has often yielded ambiguous results regarding design specifics. For instance, in the *UbiFit Garden* study, researchers found that a stylized virtual garden wallpaper effectively supported physical activity over 3 months [10], and the *BeWell* system successfully used animated sea creatures to represent sleep and social contact [12]. However, in other studies, such as *Gardy* [11], researchers found that highly stylized displays (eg, growing trees and mushrooms) were less effective than lower-stylized versions. Qualitative data from *Gardy* suggested that the issue might not have been stylization itself, but rather a lack of granularity—the inability of users to extract accurate progress data from the image. Because previous designs have varied across multiple dimensions simultaneously, it remains unclear how different dimensions of glanceable displays individually impact the understandability and usability of health feedback.

This question is particularly important in the context of smartwatch displays, which are constrained by the small screen size, the need to display time clearly first and foremost, and the very brief interactions with the feedback. To optimize the design of smartwatch-based feedback displays so that these displays can effectively support health-behavior change and be sufficiently well-liked so users are willing to keep them on their watches, we need better evidence for how different aspects of the feedback design affect how they are perceived and used.

In this paper, we report on a web-based factorial experiment with 202 participants that aimed to begin filling this knowledge gap by systematically examining the influence of 3 design dimensions—granularity of feedback, its salience, and whether the feedback is stylized—on the effectiveness of a smartwatch-based glanceable display to communicate progress on a daily step goal. Representing progress on health goals—for physical activity, sleep, diet, etc—is a common use of feedback displays on smartwatches, so the findings from our study may be applicable to other domains of health behavior change beyond physical activity.

On a methodological note, while our simulation-based study could not examine the impact of the tested design dimensions on behavior change—a research question that would require a field experiment—the size of the study, which was powered to detect small effect sizes, enabled us to examine perceptual properties and subjective judgments of the tested design dimensions with a level of rigor that could not be achieved in small-scale field deployments that are feasible for early-stage design research. As such, this study presents an example of resource-efficient optimization research that is increasingly advocated for in health and behavioral sciences [13-15].

## Methods

### Overview

To begin to understand how design affects usability and effectiveness of glanceable displays, we conducted an online simulation-based experiment that manipulated 3 design dimensions of glanceable feedback displays: stylization, salience, and granularity. In the experiment, stylization and salience were crossed in a 2×2 factorial structure, while granularity was varied across multiple levels, ranging from showing progress in 1% to 20% increments. A total of 202 Amazon Mechanical Turk (MTurk) participants were randomly assigned to 1 of 16 interface conditions. In each condition, participants were presented with a smartwatch display showing feedback on progress toward a daily step goal and were asked to estimate the level of progress that was shown.

The primary aim of the study was to investigate how the design dimensions influenced users’ accuracy in assessing goal progress displayed on a smartwatch at a glance. The primary outcome was the error in participants’ estimates of the displayed progress. A secondary aim was to examine how each design dimension affected perceived usability and acceptability.

### Rationale for the Use of an Online Simulation-Based Experiment

Given our goal of systematically evaluating the impact of design variations on users’ ability to rapidly comprehend step goal progress at a glance, we opted to conduct an online simulation-based experiment. Like any research methodology, this approach presents a set of trade-offs. Its most obvious limitation is the lack of ecological validity—the fact that participants did not interact with a real smartwatch in their natural environments. However, given our research question, this limitation is less critical than it might first appear, and it is made up for by the method’s advantages, including feasibility and scalability. Our rationale for using an online experiment was threefold.

First, the primary question addressed by this study is how variations in design influence participants’ ability to accurately perceive the level of progress toward a goal. This question concerns visual perception (ie, the ability to perceive small differences between representations) and interpretability of different types of visualization (ie, the ability to tell what the feedback display is trying to show). Such questions have routinely been studied using surveys and online experiments, including in the context of graphical representations for communicating health risks and results from medical tests [16-21]. Importantly, for our research question, an online experiment provides a *conservative* measure of the potential impact of different feedback designs, since participants are able to focus their attention on the experimental task without the distraction of other tasks that may interfere with how accurately they perceive feedback in the midst of everyday life.

Second, an online experiment enabled us to feasibly study the impacts of design with a high level of statistical rigor. Because we were concerned that small details of each design could impact performance in unforeseen ways, we created a total of 16 different interfaces. Since we expected the differences in objective performance across interfaces to be fairly small, we determined that over 200 participants would be needed to estimate these effects (power calculations are presented below), and that such a number could only be reasonably obtained by using an online platform such as Amazon MTurk. Conducting an equivalent study in the field would be prohibitively expensive, with the time and resource costs far outstripping the importance of the research question the study was trying to answer.

Third, using a simulation greatly facilitated our measurement approach. To measure user performance, we needed to compare participants’ estimates of their step goal progress to a “true” value; in addition, we needed to be able to ensure that participants were making a reasonable effort to adhere to the study procedures. Given this need for the objective “ground truth” for comparison and to know the precise state of the watch display when the participant was engaging with the experimental task, we determined that a simulation was necessary. Controlling the “glancing” behavior in the wild would be impossible, and while moderated sessions in a user testing laboratory could have been used in place of a simulation, they would not allow us to scale effectively without huge expenses that would not be merited by the expected significance of the results.

### Experimental Task

To frame the overall experiment, participants were told that they had recently bought a smartwatch, and they were using it to track their steps as they tried to increase their activity and walk 10,000 steps per day. Each experimental task was further set up with a brief scenario: participants were told that they had an important video call in a few minutes and that while they were waiting for the meeting to start, they decided to play an online game of Memory. To make sure they were not late for the meeting, they were asked to tap the spacebar a few minutes after they started playing the game to check the time, as they were told that the simulation time did not flow at the same rate as real-world time.

**Figure 1:**
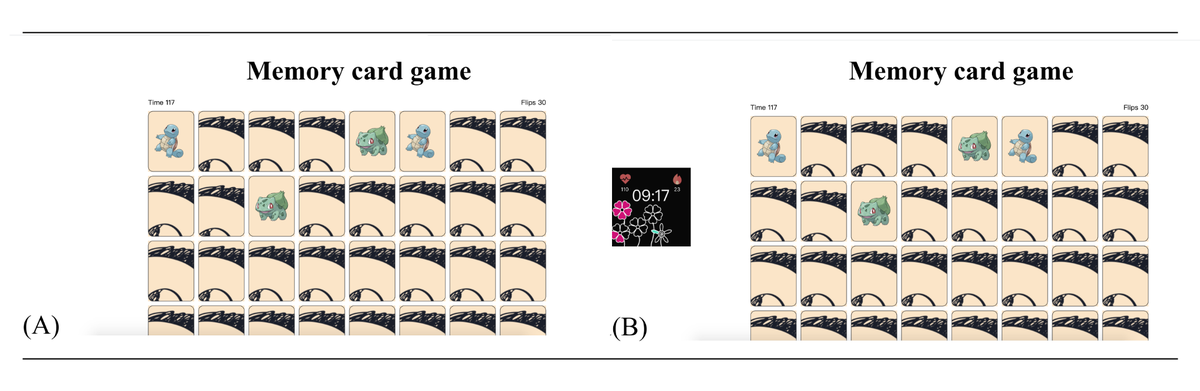
(A) Web screen without the display. (B) Web screen with the display.

Tapping the spacebar brought up an image of a smartwatch that displayed time and step-goal progress using the design for the condition to which the participant was randomized. The image of the display was sized to approximate the size of a smartwatch viewed from a foot and a half (50 cm) away, the approximate distance of the wrist from the eyes when a person looks at a watch. The smartwatch image remained on the screen for 5 seconds, after which it was hidden to mimic the act of putting the arm down (Figure 1). As soon as they saw the time and activity progress, participants could resume playing the game (ie, the game remained active even while the smartwatch display was showing) in order to mimic the return to the primary task after glancing at a watch. Overall, 15 seconds after the smartwatch display was hidden, the game was interrupted, and participants were shown a 2-question questionnaire asking them to report (1) the time they saw on the display and (2) the percentage of completion of the daily step goal shown on the watch.

### Study Procedures

All study procedures were hosted on a study website, which was linked from the task we created on the Amazon MTurk platform. When they entered the study website, participants were presented with consent information that described the study procedures, after which they were presented with a brief training that explained the display design for their assigned condition. After reading the training material, participants were given a training task where they were asked to estimate the progress on the daily step goal shown on the display that used their assigned design. After they entered their estimate, the system provided the correct answer to further assist with learning how to interpret the display accurately.

**Figure 2:**
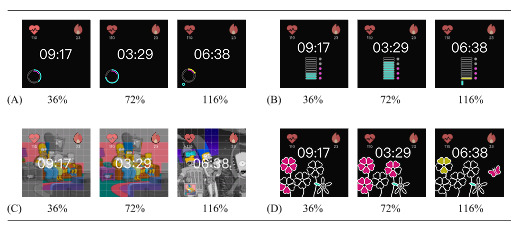
Four example displays shown at different times of day (morning, midday, and afternoon), each paired with varying levels of step-goal completion. Practice sessions were provided to help participants learn how to interpret the progress indicators. (A) Panel E in Figure 4: 1% increments. Progress turns from blue to yellow at 100%, with a small blue circle added when the goal is exceeded (eg, 116%). (B) Panel C in Figure 4: 10% increments. At 36%, 3 bars are filled. Upon overreaching the goal, the color changes to yellow and a small blue bar appears. (C) Panel N in Figure 4: 1% increments. A grayscale Simpsons image gradually regains color square by square. Exceeding the goal triggers a new image, and a small completed image appears below. (D) Panel K in Figure 4: 5% increments. Each pink flower has 5 petals; only full 5% units are shown. Beyond the goal, the flower turns yellow, and a pink butterfly appears.

Following the training, participants were presented with 3 experimental tasks. Each experimental task followed the structure outlined in the *Experimental Task* section. However, each scenario was contextualized to represent a different time of day—morning, midday, or afternoon—and featured a distinct level of step-goal completion when the smartwatch was displayed (Figure 2). After they completed all 3 experimental tasks, participants were asked to complete a questionnaire that assessed their perceptions of the usability and interpretability of the smartwatch display they used in the experiment.

**Figure 3:**
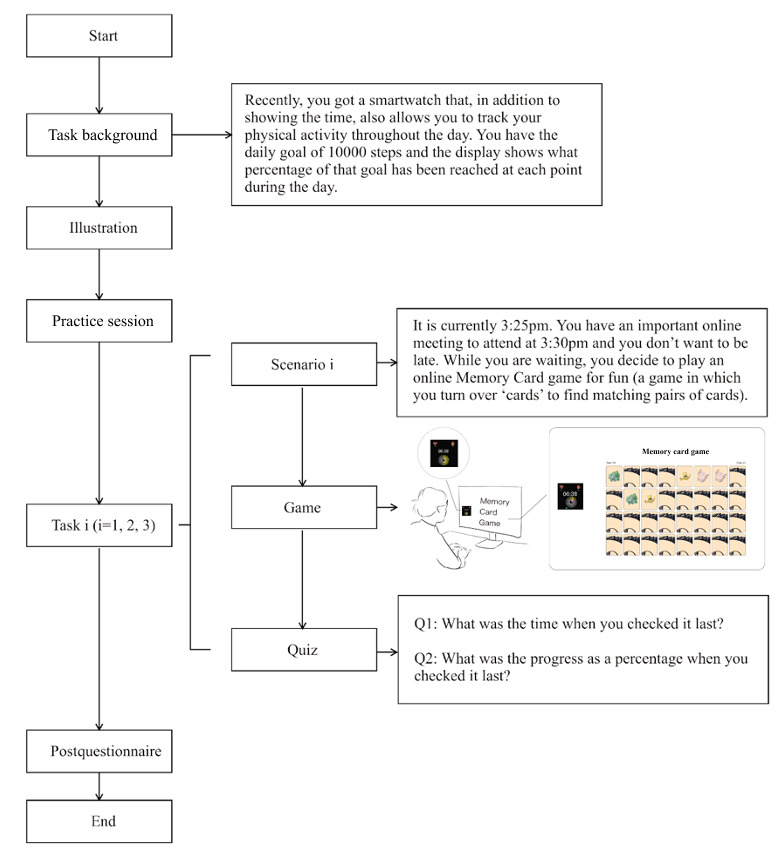
Study flow depicting participants’ progress through the training session, 3 experimental tasks, and the poststudy questionnaire.

After they submitted the questionnaire, participants were issued a code that they could enter in the MTurk task form to request payment for completing the task. Participants were compensated US $2.50 for completing the experiment. The compensation was chosen to match a US $15/hour wage, adjusted for the duration of the task (~10 minutes). The full flow of the experiment is shown in Figure 3.

### Intervention

#### Overview

**Figure 4:**
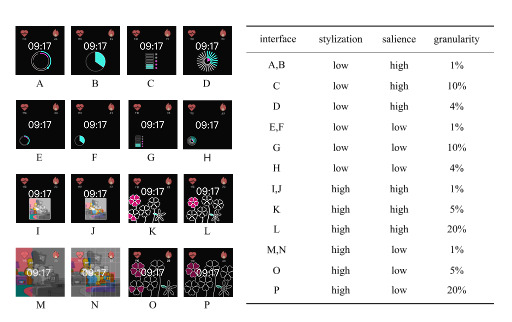
A total of 16 unique interfaces used in the study: (A) Apple Watch–inspired design using a progress circle that directly reflects the current value. (B) Apple Watch-inspired design using the pie chart that directly reflects the current value. (C) Ten horizontal bars, each representing 10% progress (eg, 36% colors 3 bars and 72% colors 7 bars). (D) Bars arranged in a circle, each representing 4% (eg, 36% colors 9 bars and 72% colors 18 bars). (E-F) Low-salience versions of panels A and B, with reduced size. (G-H) Low-salience versions of C and D, with reduced size. (I) A Simpsons figure progressively colored from left to right in 1% increments. (J) Simpsons figure divided into 100 squares; each 1% of progress colors 1 square. (K) Flower petals representing 5% each (eg, 36% of progress colors 7 petals and 72% colors 14 petals). (L) Whole flowers representing 20% each (eg, 36% of progress colors 1 flower and 72% colors 3 flowers). (M-P) Low-salience versions of panels I-L, featuring reduced saturation and brightness, along with increased transparency.

The main manipulation in the experiment was the design of the display shown to each participant. We designed 16 unique interfaces by varying 3 design dimensions: stylization (low, high), salience (low, high), and granularity (1%, 4%, 5%, 10%, 20%). Stylization and salience were manipulated at 2 levels each, while granularity varied across 5 uneven levels (Figure 4). The design dimensions tested in the experiment were as follows:

#### Stylization

Stylization refers to the use of “signs that have an intermediate degree of transparency to the signified object” [9]. In mobile health, stylized feedback is sometimes used to obscure the nature of the metrics that are being tracked, in order to display health information on displays that may be visible to other people while maintaining user privacy. We used metaphors, such as the use of flowers as iconic signs, to signify the information in the feedback displays. We chose to test stylization due to its potential to increase engagement, as high-stylized displays can be designed to be attractive and can be highly customizable [22]. In addition, high-stylized displays can be privacy-preserving, making them a good match for displaying feedback on sensitive types of health information. On the other hand, interpreting high-stylized displays requires learning, and they may not be as precise for displaying progress as graphs commonly used for data visualization. In our experiment, low-stylized displays used standard graphical formats such as bar charts, pie charts, or circles (eg, panel A in Figure 4). The selection of these charts aligns with prior research in glanceable displays, as these formats are efficient for rapid information retrieval and quick understanding on smartwatches [23-25]. High-stylized displays (eg, panels J and O in Figure 4) incorporated more expressive visuals, including cartoon characters and flowers. Inspired by the customizable cartoon interfaces on the Apple Watch, such as Snoopy, we chose pictures from “*The Simpsons*”—a widely watched cartoon in the United States—to represent progress in the high-stylized condition. Additionally, drawing inspiration from the *Ubifit Garden* [26], we used flowers as an alternative high-stylized representation to design a visually engaging form of feedback.

#### Granularity

Granularity refers to the size of the increment used to display progress. High-granularity feedback can represent small changes (eg, each 1% of goal attainment), while low-granularity feedback represents larger increments (eg, every 10% or 20% of progress). Due to the small size of the smartwatch screen and the need to display multiple pieces of information, including time, designers may be tempted to design smartwatch feedback that uses lower data granularity in order to satisfy aesthetic considerations and feedback readability. On the other hand, high-granularity feedback has been shown to aid progress estimation and increase physical activity motivation [27,28].

To evaluate these effects, we selected 5 granularity levels: 1%, 4%, 5%, 10%, and 20%. These levels are not evenly spaced, as they are exploratory design choices based on our design inspirations and established user interface paradigms in the wearable market. The 1% granularity level represents the high-precision displays found in Apple Watch and Garmin designs, where progress is mapped to exact pixel or degree movements. The 4%, 5%, and 10% levels reflect common progress bar increments found in Fitbit interfaces (eg, progress lights in the Fitbit Flex), where progress is often discretized into “steps” or blocks to enhance glanceability. Finally, the 20% level was inspired by HCI designs such as the *UbiFit Garden* [26], which uses discrete icons (eg, 1 flower) to represent the completion of a significant sub-goal. We chose the 20% step for this purpose as it represents a moderately large increment (ie, 1/5th) commonly used to represent goal progress.

#### Salience

Salience refers to the degree to which a stimulus captures attention by standing out from its environment. Rather than being a single attribute, salience is a composite of visual factors—such as contrast, size, and positioning—that determine how much “attentional grab” a display exerts [29-31]. Salience is known to influence the perception of peripheral displays [32], yet operationalizing this concept for smartwatch interfaces proved nontrivial, as different design styles required different levers to manipulate user attention.

For low-stylized displays, which used standard graphs, we operationalized salience primarily through size and chart position [29]. High-salience interfaces featured progress feedback centered on the screen at a height exceeding 100 points, demanding immediate focus. Conversely, low-salience versions used a complication style—placed in the screen’s corner at a height of 60 points—following Apple Watch Human Interface Guidelines. Here, the reduced size and peripheral placement minimized the display’s ability to easily compete for the user’s attention vs the time display.

The operationalization became more nuanced for high-stylized interfaces, where simply shrinking the designs would have rendered them unreadable due to their visual complexity. Instead, salience was manipulated by varying the relationship between the representation and the background. The high-salience condition used a centrally positioned 128-point display that remained a distinct, foreground element (eg, panel I in Figure 4). In contrast, the low-salience condition used a 300-point display that covered the entire screen but was designed to recede into the background (eg, panel M in Figure 4). By reducing the saturation and brightness while increasing transparency, this larger display was made less visually prominent to avoid distracting from the watch’s primary timekeeping function.

### Randomization

When they entered the experiment, each participant was automatically randomized, with the probability of 1 out of 16 (6.25%), to 1 of the 16 experimental designs of progress feedback. All instructions for interpreting the display, the test session at the start of the experiment, as well as each of the 3 experimental tasks used the feedback design to which the participant was randomized.

Each participant completed 3 experimental tasks that used different levels of step-goal progress: 36%, 72%, and 116%. The numbers were chosen to represent low, moderate, and goal-met-and-exceeded progress on the daily step goal, and to not be round, easily guessable numbers. To control for order effects, the order of the 3 tasks was randomized for each participant. This meant that, for instance, a participant may see the interface that represents 116% goal attainment first, followed by interfaces that show 36% and 72% goal attainment. Each level of progress was shown to each participant only once.

### Measures

The objective of our study was to find out how the tested design dimensions affect the ability of participants to accurately assess the smartwatch progress feedback display at a glance. Consequently, our primary outcome was the absolute error of the participants’ progress estimates, calculated as the absolute difference between the progress estimate and the true progress value, which allowed us to assess the overall accuracy of participants’ ratings without taking into consideration whether their ratings were above or below the true value of the goal progress.

Our secondary outcomes included the self-reported ratings of the displays’ usability and usefulness. These were assessed using a questionnaire that participants completed after they completed all 3 experimental tasks. The questionnaire was based on the System Usability Scale [33] and a questionnaire used by Shami et al [34] to assess the perceived strengths and weaknesses of glanceable displays. The constructs assessed and the questions used are shown in Textbox 1. Questions Q6-Q7 and Q12-Q14 were adapted from the System Usability Scale; questions Q1-Q5 and Q8-Q11 (except Q10) were adapted from Shami et al [34]. All questions were assessed on a 5-point Likert scale that ranged from *strongly disagree* to *strongly agree*. Q10 was an attention check question that we used to filter out Amazon MTurk workers who were answering questions without paying even minimal attention.

**Poststudy questionnaire. Constructs and corresponding question items are listed below.**

**Noticeability**

- Q1: I could easily notice the progress display.
- Q2: I could notice the change in my progress across 3 scenarios.

**Comprehension**

- Q3: I felt confident in being able to understand the information regarding my progress.
- Q4: I felt confident in being able to understand the information in the progress display just by glancing at it.

**Relevance**

- Q5: The display provided me with the information I needed to track my progress.

**Complexity**

- Q6: The smartwatch display showed too much distracting information.
- Q7: I found the smartwatch display unnecessarily complex.

**Division of attention**

- Q8: I was able to maintain focus on playing the game while checking the time and my progress.
- Q9: I was able to shift my attention between playing the game and checking the smartwatch display easily.

**Attention check**

- Q10: Please skip this question and move to the next one. This is just to screen out random clicking.

**Attractiveness**

- Q11: I found the design of the smartwatch display attractive.

**Usability**

- Q12: I think the way the progress was shown is useful.
- Q13: I think that I would like to use this smartwatch display on an ongoing basis.
- Q14: I would imagine that most people would learn to use this system very quickly.

### Statistical Analysis

#### Sample Size and Power

The study was powered to detect the main effects of the 3 design dimensions on the primary outcome, the absolute value of the error in progress ratings. While we could not find any existing literature that would readily provide data on the sizes and distributions of the likely effects for the measure we were interested in, we started with the data from the work of Gouveia et al, who designed 21 glanceable displays for showing activity progress [1]. To derive a functional baseline, we examined their *Goal Completion* condition (mean 5340, SD 4528), which emulates established commercial standards (eg, Fitbit Flex), and compared it against their most divergent design, the *Gardy* condition (mean 3760, SD 3511). Using the pooled standard deviation method, this comparison yielded an estimated effect size of approximately *d*=0.39. However, to remain conservative and ensure the study could detect even subtler differences between our own design iterations, we assumed a small effect size of *d*=0.2. Further, we assumed a moderate, 65% correlation between repeated measures. With 90% power and the alpha level set to .05, the required sample size was 204.

#### Analysis

For the analyses of the progress estimation error rates, we used the generalized estimating equation (GEE), which is an extension of linear regression that takes into account the nested nature of repeated-measure data. Given that stylization and salience had 2 levels, but granularity had 5 levels, we estimated the main effects for stylization and salience in a single model, as is typically done in factorial experiments, but we fit a separate model to estimate the effect of granularity. To improve model fit, in all analyses, the estimated error was square-root transformed—that is, the dependent variable was defined as:

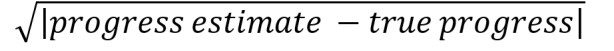



Diagnostic plots for the GEE model were assessed to ensure valid statistical inference. The observed striations in the residuals vs fitted plot were confirmed to be artifacts of the discrete, integer-based nature of the raw data rather than model misspecification. The residual distribution confirms that the model appropriately accounts for the data structure.

To examine responses from the poststudy questionnaire, we first created scores for each construct measured by the questionnaire (ie, noticeability, comprehension, relevance, complexity, division of attention, attractiveness, and usability) by averaging each participant’s answers to the questions for that construct. We then fitted linear regression models that used these construct scores as dependent variables and indicators for the design dimensions as predictors.

In line with best practices for factorial experiments [35], models analyzing the stylization and salience design dimensions used effect coding to code design-dimension indicators. With effect coding, 1 level of a factor (eg, high-salience) is coded as +1, and the other level of the factor (eg, low-salience) is coded as –1 in the models. The use of effect coding enables a marginal interpretation of the main effects for each factor: if the *β* coefficient for a particular design dimension (eg, salience) is found to be significant, that finding can be interpreted as indicating that that design dimension has an impact on the dependent variable (accuracy of progress estimation, usability, etc) averaging across other tested factors and other measured and unmeasured influences.

### Eligibility, Recruitment, and Data Integrity Verification

Recruitment was done through the Amazon MTurk platform. We created an MTurk task that linked to the study website that hosted the experiment. Eligible participants were required to be 18 years of age or older and could take part in the experiment only once. Attempts at participating more than once were recognized based on the MTurk worker ID and were filtered out prior to the data analysis.

To ensure high data quality—MTurk workers sometimes click through a task to get paid without actually engaging with the task [36,37]—prior to conducting the experiment, we developed 2 attention checks that we used as criteria for *a priori* exclusion of participants from the dataset. The first check used a metric called string edit distance [38], which measures the similarity of 2 strings in terms of the number of operations (insertions, deletions, etc) that are required to transform one string into the other. In our pilot work, we found that the edit distance of 2 effectively identified responses to the goal-progress estimation that were clearly fallacious (eg, the same number was provided as an answer to all 3 experimental tasks). We, therefore, removed from the analytical data set all participants for whom the edit distance between their estimates of goal progress and the correct answer was greater than or equal to 2. The second exclusion criterion used the attention check question in the poststudy questionnaire (Q10 in Textbox 1). The question asked participants to skip the question and leave it unanswered. Participants who answered the question (ie, who clicked on any radio button on the Likert scale for that question) were excluded from the dataset.

The study protocol was approved by our university’s Behavioral and Social Sciences Institutional Review Board.

## Results

### Participants Sample

We recruited 400 participants on Amazon MTurk, targeting adults who were interested in smartwatches and physical activity. Participants were recruited in 4 waves of 100 participants each. Each wave of recruitment was followed by the exclusion of participants who violated one of the criteria for data integrity validation. We would then start the next wave if the required number of participants had not been reached.

After applying data integrity criteria, 202 participants remained in the final dataset. Of these, 35.64% (n=72) identified as female. In terms of smartwatch usage, 29.2% (n=59) were current users, 38.61% (n=78) were former users, and 32.18% (n=65) had never used a smartwatch. The age distribution was skewed toward younger and middle-aged adults. The largest age group was 25-34 years (n=87, 43.07%), followed by 35-44 years (n=57, 28.21%) and 45-54 years (n=33, 16.34%). Participants aged 18-24 years accounted for 6.93% (n=14) of the sample, while those aged 55-64 years and older comprised 3.47% (n=7) and 1.98% (n=4), respectively.

### Stylization Negatively Affects the Accuracy of Progress Assessment

Table 1 presents the results of the GEE analysis examining the effects of salience and stylization on the accuracy of participants’ goal-progress estimates. Since the outcome variable was square-root transformed, the reported *β* coefficients should be squared to interpret effects on the original scale. To make the results interpretable, we note that the median absolute error across all conditions was 14 (IQR 4-47) points. Our findings indicate that stylization significantly reduces the accuracy of progress assessments. Compared to low-stylized representations, high-stylized ones led to an increase in error by 4.52 points, rising from 8.98 for low-stylized conditions to 13.50 points for high-stylized conditions. We found no significant impact for salience, however.

**Table 1:** Generalized estimating equation model results examining the effects of stylization, salience, age, gender, and user status on the accuracy of progress estimates (square-root transformed outcome).

	Coefficient	SE	*z*	*P* value	95% CI
Intercept	3.335	0.529	6.307	<.001	2.299 to 4.372
Stylization	0.339	0.122	2.764	.006	0.098 to 0.579
Salience	–0.011	0.121	–0.090	.93	–0.248 to 0.226
Age	0.021	0.012	1.783	.07	–0.002 to 0.044
Gender	0.049	0.124	0.391	.70	–0.195 to 0.292
Current user	0.256	0.336	0.763	.45	–0.402 to 0.914
Previous user	0.976	0.287	3.395	.001	0.412 to 1.539

### Nonlinear Effects of Granularity on Estimated Absolute Error

Table 2 presents the results of a GEE analysis examining the effects of salience, stylization, and granularity on the accuracy of participants’ goal-progress estimates. By including all 3 factors in a single model, we isolated the marginal impact of each variable. In this combined linear model, granularity demonstrated a marginally significant independent effect on estimation error (*P*=.08). However, because granularity was unevenly distributed across feedback designs—with half of the panels using the finest granularity level (1%)—and preliminary diagnostics suggested a nonlinear relationship, we conducted a follow-up analysis. As reported in Table 3, we used a model specifically parameterized with a quadratic term to account for this complexity. These results revealed a U-shaped relationship: error decreased as granularity *decreased*, up to a point, after which further decreases in granularity led to higher error.

**Table 2:** Generalized estimating equation model results examining the effects of stylization, salience, granularity, age, gender, and user status on the accuracy of progress estimates (square-root transformed outcome).

	Coefficient	SE	z	*P* value	95% CI
Intercept	3.230	0.541	5.973	<.001	2.170 to 4.290
Stylization	0.311	0.124	2.507	.01	0.068 to 0.554
Salience	–0.007	0.121	–0.056	.96	–0.248 to 0.226
Granularity	0.033	0.019	1.732	.08	–0.004 to 0.070
Age	0.020	0.012	1.652	.10	–0.004 to 0.043
Gender	0.034	0.124	0.272	.79	–0.210 to 0.278
Current user	0.254	0.335	0.759	.45	–0.402 to 0.911
Previous user	0.961	0.283	3.396	.001	0.406 to 1.515

**Table 3:** Generalized estimating equation model results examining the nonlinear effects of granularity on the accuracy of progress estimates (square-root transformed outcome).

	Coefficient	SE	*z*	*P* value	95% CI
Intercept	4.599	0.214	21.465	<.001	4.179 to 5.019
Granularity	–0.114	0.071	–1.599	.11	–0.254 to 0.026
Granularity squared	0.008	0.003	2.437	.015	0.002 to 0.015

We examined 5 levels of feedback display granularity: 1%, 4%, 5%, 10%, and 20%. Given that the outcome variable was square-root transformed, predicted values were squared to interpret error on the original scale. Decreasing granularity from 1% to 4% reduced the predicted error from 20.19 to 18.24, a decrease of 1.95 points. A further decrease in granularity from 4% to 5% resulted in a smaller reduction of 0.36 points, lowering the predicted error to 17.88 at the 5% granularity level. Transitioning from 5% to 10%, the error began to increase slightly, rising by 0.26 points to 18.14 at 10% granularity. When granularity decreased from 10% to 20%, the predicted error sharply increased by 12.32 points, reaching 30.46. These findings reveal a U-shaped relationship between granularity and error, suggesting that when feedback is presented on smartwatch-sized screens, moderate levels of granularity enhance accuracy, whereas high granularity and overly coarse representations hinder users’ ability to estimate goal progress correctly.

According to the model, the optimal granularity level is around 7.1%. Since this value was not directly tested in our feedback designs, it suggests that the lowest predicted error would occur at a granularity level of 5% or 10%.

### Stylization Negatively Affects Perception of Comprehension, Relevance, Complexity, Attention, Attractiveness, and Usability Ratings

As shown in Textbox 1, we grouped the subjective perception items from the postquestionnaire into 7 categories: noticeability, comprehension, relevance, complexity, division of attention, attractiveness, and usability. To examine the impact of stylization and salience on participants’ subjective perceptions of the feedback displays, we conducted linear regression analyses.

Tables 4-9 present the results of the linear regression analyses examining the effects of stylization and salience on participants’ perceptions of the interfaces, including comprehension, relevance, complexity, division of attention, attractiveness, and usability (see Table S1 in Multimedia Appendix 1 for the impact of stylization on noticeability). Perceptions were measured using a 5-point Likert scale across 7 categories. Stylization was found to negatively influence 6 of the 7 dimensions of user perceptions: comprehension, relevance, complexity, attention, attractiveness, and usability. When using high-stylized feedback designs, participants reported greater difficulty comprehending the information (–0.486 points; *P*=.003), perceived the interface as more complex (–0.690 points; *P*<.001), and felt that less relevant information was provided (–0.476 points; *P*=.002). Additionally, they found it harder to maintain focus or shift attention between the interface and the game (–0.378 points; *P*=.022), and rated the interfaces as less attractive (–0.338 points; *P*=.047) and less usable (–0.532 points; *P*=.001).

**Table 4:** Linear regression analysis of the effects of stylization and salience on comprehension scores (reference group: people who have never used a smartwatch before).

	Coefficient	SE	*t* test (*df*)	*P* value	95% CI
Intercept	3.415	0.339	10.069 (195)	<.001	2.746 to 4.084
Stylization	–0.243	0.079	–3.060 (195)	.003	–0.400 to –0.086
Salience	0.146	0.078	1.886 (195)	.06	–0.007 to 0.300
Age	–0.005	0.008	–0.701 (195)	.48	–0.021 to 0.010
Gender	0.116	0.082	1.419 (195)	.16	–0.045 to 0.278
Current user	0.317	0.198	1.602 (195)	.11	–0.073 to 0.708
Previous user	0.351	0.190	1.846 (195)	.07	–0.024 to 0.727

**Table 5:** Linear regression analysis of the effects of stylization and salience on relevance scores (reference group: people who have never used a smartwatch before).

	Coefficient	SE	*t* test (*df*)	*P* value	95% CI
Intercept	3.460	0.321	10.775 (195)	<.001	2.827 to 4.093
Stylization	–0.238	0.075	–3.170 (195)	.002	–0.387 to –0.090
Salience	0.103	0.074	1.402 (195)	.16	–0.042 to 0.248
Age	0.001	0.007	0.198 (195)	.84	–0.013 to 0.016
Gender	0.137	0.078	1.762 (195)	.08	–0.016 to 0.290
Current user	0.222	0.187	1.182 (195)	.24	–0.148 to 0.591
Previous user	0.283	0.180	1.572 (195)	.12	–0.072 to 0.639

**Table 6:** Linear regression analysis of the effects of stylization and salience on complexity scores (reference group: people who have never used a smartwatch before).

	Coefficient	SE	*t* test (*df*)	*P* value	95% CI
Intercept	3.004	0.393	7.644 (195)	<.001	2.229 to 3.779
Stylization	–0.345	0.092	–3.745 (195)	<.001	–0.526 to –0.163
Salience	0.031	0.090	0.339 (195)	.73	–0.147 to 0.208
Age	0.004	0.009	0.426 (195)	.67	–0.014 to 0.021
Gender	0.084	0.095	0.885 (195)	.38	–0.103 to 0.272
Current user	0.019	0.229	0.084 (195)	.93	–0.433 to 0.472
Previous user	–0.754	0.221	–3.417 (195)	.001	–1.189 to –0.319

**Table 7:** Linear regression analysis of the effects of stylization and salience on division of attention scores (reference group: people who have never used a smartwatch before).

	Coefficient	SE	*t* test (*df*)	*P* value	95% CI
Intercept	2.750	0.350	7.849 (195)	<.001	2.059 to 3.441
Stylization	–0.189	0.082	–2.309 (195)	.022	–0.351 to –0.028
Salience	0.136	0.080	1.701 (195)	.09	–0.022 to 0.295
Age	0.003	0.008	0.320 (195)	.75	–0.013 to 0.018
Gender	0.104	0.085	1.227 (195)	.22	–0.063 to 0.271
Current user	0.559	0.204	2.733 (195)	.007	0.156 to 0.962
Previous user	0.874	0.197	4.444 (195)	<.001	0.486 to 1.262

**Table 8:** Linear regression analysis of the effects of stylization and salience on attractiveness scores (reference group: people who have never used a smartwatch before).

	Coefficient	SE	*t* test (*df*)	*P* value	95% CI
Intercept	3.286	0.361	9.093 (195)	<.001	2.573 to 3.998
Stylization	–0.169	0.085	–2.001 (195)	.047	–0.336 to –0.002
Salience	0.007	0.083	0.082 (195)	.93	–0.156 to 0.170
Age	–0.001	0.008	–0.141 (195)	.89	–0.017 to 0.015
Gender	0.074	0.087	0.849 (195)	.40	–0.098 to 0.247
Current user	0.163	0.211	0.773 (195)	.44	–0.253 to 0.579
Previous user	0.367	0.203	1.812 (195)	.07	–0.033 to 0.767

**Table 9:** Linear regression analysis of the effects of stylization and salience on usability scores (reference group: people who have never used a smartwatch before).

	Coefficient	SE	*t* test (*df*)	*P* value	95% CI
Intercept	3.369	0.324	10.409 (195)	<.001	2.730 to 4.007
Stylization	–0.266	0.076	–3.505 (195)	.001	–0.415 to –0.116
Salience	0.110	0.074	1.487 (195)	.14	–0.036 to 0.256
Age	–0.010	0.007	–1.331 (195)	.18	–0.024 to 0.005
Gender	0.102	0.078	1.301 (195)	.19	–0.053 to 0.256
Current user	0.512	0.189	2.709 (195)	.007	0.139 to 0.884
Previous user	0.566	0.182	3.118 (195)	.002	0.208 to 0.924

### 10% Granularity Yields the Most Favorable Perceptions

Compared to modeling granularity as a continuous predictor, treating it as a categorical variable provided a clearer understanding of how different granularity levels impacted subjective responses. Tables 10-12 present the results of linear regression analyses examining the effects of granularity on participants’ perceptions of the interfaces, including comprehension, complexity, and division of attention (see Tables S2-S5 in Multimedia Appendix 1 for the impact of granularity on noticeability, relevance, attractiveness, and usability).

**Table 10:** Linear regression analysis of the effects of granularity on comprehension scores (reference group: 1% granularity).

	Coefficient	SE	*t* test (*df*)	*P* value	95% CI
Intercept	3.435	0.110	31.132 (197)	<.001	3.217 to 3.653
Granularity=4%	0.065	0.230	0.283 (197)	.78	–0.388 to 0.518
Granularity=5%	–0.435	0.265	–1.642 (197)	.10	–0.957 to 0.087
Granularity=10%	0.656	0.222	2.961 (197)	.003	0.219 to 1.093
Granularity=20%	–0.046	0.283	–0.163 (197)	.87	–0.603 to 0.511

**Table 11:** Linear regression analysis of the effects of granularity on complexity scores (reference group: 1% granularity).

	Coefficient	SE	*t* test (*df*)	*P* value	95% CI
Intercept	2.810	0.133	21.067 (197)	<.001	2.547 to 3.073
Granularity=4%	0.223	0.278	0.804 (197)	.42	–0.324 to 0.771
Granularity=5%	–0.429	0.320	–1.340 (197)	.18	–1.060 to 0.202
Granularity=10%	0.766	0.268	2.860 (197)	.005	0.238 to 1.294
Granularity=20%	0.107	0.342	0.312 (197)	.76	–0.567 to 0.780

**Table 12:** Linear regression analysis of the effects of granularity on division of attention scores (reference group: 1% granularity).

	Coefficient	SE	*t* test (*df*)	*P* value	95% CI
Intercept	3.295	0.117	28.121 (197)	<.001	3.064 to 3.526
Granularity=4%	0.305	0.244	1.250 (197)	.21	–0.176 to 0.786
Granularity=5%	–0.390	0.281	–1.387 (197)	.17	–0.945 to 0.164
Granularity=10%	0.523	0.235	2.224 (197)	.027	0.059 to 0.987
Granularity=20%	0.038	0.300	0.128 (197)	.90	–0.553 to 0.630

Among the 5 granularity levels (1%, 4%, 5%, 10%, and 20%), the 10% granularity level yielded the most favorable perceptions. Relative to the 1% granularity group, participants in the 10% group reported the interface as easier to comprehend (+0.656 points; *P*=.003), less complex (+0.766 points; *P*=.005), and easier to maintain focus on (+0.523 points; *P*=.03).

To further evaluate the performance of the 10% group, we reran the analysis using the 10% group as the reference category (Tables S6-S12 in Multimedia Appendix 1). Consistent with the previous comparison, we found that participants in all other granularity groups rated the feedback designs as harder to comprehend (1%: –0.656; *P*=.003; 4%: –0.591; *P*=.04; 5%: –1.091; *P*<.001; 20%: –0.702; *P*=.03). Participants in the 1% (–0.766 points; *P*=.005) and 5% (–1.195 points; *P*=.002) groups also perceived the feedback designs as more complex compared to the 10% group. Similarly, participants in the 1% (–0.523 points; *P*=.03) and 5% (–0.913 points; *P*=.006) groups found it more difficult to maintain focus, compared to the 10% group. Additionally, compared to the 10% group, participants in the 5% group perceived the feedback design as harder to notice changes in (–0.634 points; *P*=.024), less informative (–0.779 points; *P*=.009), and less useful overall (–0.848 points; *P*=.006).

### Effects of Smartwatch Experience on Accuracy and Subjective Perceptions

Prior smartwatch experience was associated with decreased accuracy in progress assessment. As shown in Table 1, compared to people who have never used a smartwatch before, being a previous smartwatch user—but not a current user—increased the error by approximately 7.46 points, with the error rising from 11.13 to 18.59 points (median absolute error 14, IQR 4-47 points).

Despite exhibiting lower accuracy in progress estimation compared to participants who had never used a smartwatch, previous smartwatch users reported more favorable subjective perceptions across several measures. As seen in Tables 6, 8, and 9, compared to participants who had never used a smartwatch, previous smartwatch users found the interface easier to maintain focus on (+0.874 points, *P*<.001) and more useful (+0.566 points, *P*=.002). However, they also perceived the feedback displays as more complex, rating them 0.754 points lower in simplicity *P*=.001). In Table 13, compared to current smartwatch users, previous smartwatch users also perceived the feedback design as harder to notice changes in (–0.773 points, *P*=.001).

**Table 13:** Linear regression analysis of the effects of stylization and salience on noticeability scores (reference group: current smartwatch user).

	Coefficient	SE	*t* test (*df*)	*P* value	95% CI
Intercept	3.253	0.407	8.003 (195)	<.001	2.452 to 4.055
Stylization	–0.689	0.184	–3.745 (195)	<.001	–1.052 to –0.326
Salience	0.061	0.180	0.339 (195)	.73	–0.294 to 0.416
Age	0.004	0.009	0.426 (195)	.67	–0.014 to 0.021
Gender	0.168	0.190	0.885 (195)	.38	–0.207 to 0.544
Never used user	–0.019	0.229	–0.084 (195)	.93	–0.472 to 0.433
Previous user	–0.773	0.224	–3.449 (195)	.001	–1.215 to –0.331

Current smartwatch users did not show significant differences in accuracy compared to participants who had never used a smartwatch, but reported more positive subjective perceptions. Specifically, as seen in Table S1 in Multimedia Appendix 1, Tables 7 and 9, they rated the feedback designs as easier to notice changes in (+0.354 points; *P*=.053), easier to maintain or shift attention (+0.559 points; *P*=.007), and more useful (+0.512 points; *P*=.007), all on a 5-point scale.

## Discussion

### Principal Results

Our study examined how variations across 3 design dimensions—stylization, salience, and granularity—influenced participants’ ability to interpret glanceable smartwatch feedback displays.

Results showed that high stylization negatively affected the accuracy of progress assessments. Regarding granularity, levels of 5% and 10% could be seen as a sweet spot, resulting in lower interpretation errors. Participants in the 10% granularity group also reported more favorable subjective perceptions compared to those in other granularity conditions. Salience did not influence either the accuracy of interpreting progress or participants’ subjective perceptions.

Interestingly, participants who had previously used a smartwatch but were no longer actively using one performed worse in assessing progress compared to those who had never used a smartwatch. However, both previous and current smartwatch users reported more positive subjective evaluations of feedback displays than participants who had never used a smartwatch.

### Increased Interpretation Errors Caused by Stylization Present Design Trade-Offs

Our results indicate that high stylization negatively affected interpretation accuracy, increasing progress errors by 4.52 points. This finding should be considered alongside several factors that may influence the real-world viability of stylized displays. First, the observed accuracy gap might narrow with increased familiarity; while our study used a brief practice period, long-term use of a specific stylized interface could result in users learning to better interpret its visual patterns, potentially improving performance over time. Second, stylization may serve as a privacy-preserving layer, offering users a degree of discretion by preventing onlookers from easily discerning personal data. Third, the aesthetic qualities of such interfaces might boost user engagement and check frequency, which could enhance overall effectiveness [22,39,40].

In contrast, low-stylized displays are preferred when feedback precision is critical for decision-making. This is particularly relevant in safety-critical contexts or data-driven health management, such as monitoring blood glucose levels [41] or tracking caloric balance. In these scenarios, the requirement for high levels of precision makes stylized feedback a suboptimal choice. Ultimately, the use of stylization involves a trade-off between the necessity for interpretative accuracy and the user’s potential preference for discretion and aesthetic engagement.

### Finding the Sweet Spot in Granularity for Glanceable Displays

We found that the relationship between granularity and accuracy in progress assessment is nonlinear. Accuracy initially increases as granularity decreases (eg, from 1% to 5%), but the rate of increase slows down between 5% and 10% and then reverses direction. This pattern indicates the presence of an inflection point—an optimal granularity level, estimated at 5%-10% in our data—at which accuracy peaks. Beyond this point, accuracy declines as granularity increases further.

These results are not completely unexpected, as there are situations where high levels of feedback granularity may not be helpful. For instance, in very small feedback displays, such as smartwatch complications, feedback granularity can quickly exceed users’ ability to visually discriminate between states of the display. In such situations, a lower-granularity display may more effectively convey changes in the state through more easily perceived jumps than a high-granularity display. Similarly, in many contexts, there are limits to the level of granularity that is needed for decisions. For instance, Hawley et al [16] and Zikmund-Fisher et al [20] found that variations in the granularity of the graphs about the risk of taking different medications did not have a significant impact on people’s choices of which medicine to take. The researchers used n-in-100 (ie, among 100 people, the number of people who have side effects) as low granularity and n-in-1000 as high granularity. Their results indicated that when the level of granularity met the upper limit of the demand, participants’ assessments became unaffected by further increases in granularity.

Our result shows that the sweet spot for goal-progress feedback displays may be at 5% or 10% granularity, which aligns with previous research. For instance, Kong et al [42] observed that participants estimating the proportion between 2 rectangles (displayed in a constrained space) tended to round their answers to multiples of 5. Our results showed the same tendency—participants frequently reported progress estimates in multiples of 5. This suggests that interfaces designed with 5% or 10% granularity may support faster and more accurate assessments, as users can more easily map perceived progress to familiar reference points.

Taken together, these results suggest that pursuing very high granularity does not necessarily improve users’ ability to notice changes or make informed decisions. Conversely, very low granularity may increase errors in progress assessment and fall short in contexts where greater precision is required [41]. A granularity level of 5% or 10% appears to strike an effective balance—supporting precision, enabling users to notice changes, and reducing estimation errors.

### Prior Use Followed by Abandonment May Reflect Interpretation Difficulties

Lastly, our results indicate that previous smartwatch users made more errors in perceiving progress compared to those who had never used a smartwatch. Specifically, their error was 7.46 points higher—representing the largest effect on interpretation accuracy observed in our study. While we are not sure why this discrepancy exists, one intriguing possibility is that people who are less effective at interpreting information displayed on smartwatches are more likely to abandon them. This possibility might be reflected in participants’ subjective perceptions. Compared to individuals who have never used a smartwatch, previous smartwatch users rated the feedback display as more complex (–0.754 points; *P*=.001; Table 6). Even when compared to current smartwatch users, previous smartwatch users still perceived the feedback display as more complex (–0.773 points; *P*=.001; Table 13). However, as shown in Table 7 and Table 9, previous smartwatch users also found the interface more useful (+0.566 points; *P*=.002; Table 9) and reported it was easier to maintain focus while using it (+0.874 points; *P*<.001; Table 7). Current smartwatch users similarly rated the feedback display as more useful (+0.512 points; *P*=.007; Table 9) and easier to focus on (+0.559 points; *P*=.007; Table 7). Notably, current users did not report any statistically significant negative perceptions of the feedback display interface.

If this is the case, researchers who develop smartwatch-based interventions may want to use prior smartwatch use as a tailoring variable for providing additional training or using alternative forms of intervention feedback. Investigating how predictive this variable is for other intervention effects could be an interesting avenue for future research.

### Challenges in Operating Design Dimensions From Theories to Practices

Our experience in constructing these interfaces suggests a broader challenge regarding how to cleanly operationalize design dimensions when transitioning from theories to practical applications. While design dimensions such as granularity, stylization, and salience are conceptually distinct, their implementation in a real-world context like smartwatch displays may be inherently constrained by practical requirements.

For instance, the tension between exact estimations and low-granularity designs reflects the trade-off between accuracy and interpretability. For glanceable displays, while providing accurate data is important, designs need to remain interpretable to be efficiently accessed under cognitive load. While asking participants to estimate the range of progress might have aligned more naturally with low-granularity interfaces, using an exact estimation task across all conditions allowed us to observe how different designs influence this cognitive balance. This indicates that practical constraints can make it difficult to cleanly separate a single design dimension from its functional impact on performance. Another operationalization challenge for stylization and granularity is further discussed in the Limitations section.

These observations suggest that design dimensions that are theoretically distinct are often not orthogonal in practice. Operationalizing a single dimension in isolation, without accounting for these functional interdependencies, risks producing interfaces that are either visually incoherent or practically irrelevant.

### Limitations

#### Methodological Limitations

As we note in the Method section, the main trade-off of our decision to use an online experiment involves sacrificing some ecological validity for the increased statistical rigor and feasibility of studying many design variations for feedback displays. One other notable limitation of the study is that it did not allow us to study additional questions about the influence of time and attentional context on display interpretation. In real-world use, over time, users are likely to learn how to interpret any (reasonable) type of feedback display. So, it is possible that the differences in interpretation accuracy we found in our study for unfamiliar displays would gradually decrease with repeated use. Similarly, how accurately a person interprets a feedback display at any given time is likely to have to do in part with why they are looking at the watch. If the person is in a hurry and just wants to see what time it is, they may not notice the feedback display at all. If they are trying to check goal progress, they would pay closer attention to the display. While our study was closer to the latter setting, carefully studying how exactly time and context affect feedback interpretation would require a different study design. Whether a simulation could still be used to study those processes is an interesting question that would be worthwhile for design researchers to consider. The answer will likely depend on the exact processes that are being studied and whether a simulation could incorporate the key factors hypothesized to influence those processes. Determining this involves thinking carefully about the causal processes that underlie the phenomenon of interest.

#### Design Space Constraints

Another limitation of this work concerns the interdependence of the design dimensions in practice. While we conceptually defined granularity, stylization, and salience as distinct factors, they were not always perfectly decoupled in our experimental stimuli. For instance, changes in granularity were occasionally tied to shifts in representation type, such as transitioning from a circular progress ring to a segmented bar chart to accommodate higher data resolution.

This confounding reflects a considered trade-off between experimental control and the practical applicability of the stimuli. Given the exploratory nature of mapping this design space, we aimed to present participants with functional and coherent interfaces that mirror the types of glanceable displays that can be used in everyday contexts. Strictly isolating a single dimension by increasing granularity without adjusting the visual representation could have resulted in artifacts that were visually awkward or practically illegible. While this approach prioritized the holistic integrity of the designs, we acknowledge that it limits our ability to isolate the independent impact of each individual dimension. Future work could build on these findings by employing more constrained experimental setups to further disentangle these interconnected factors.

### Conclusions

In this paper, we presented a study that tested 16 glanceable feedback displays to examine the effects of 3 design dimensions—stylization, salience, and granularity—on the accuracy of progress assessment. Our findings show that stylization negatively affected both the accuracy of assessing goal progress and perceptions of comprehension, relevance, complexity, attention, attractiveness, and usability. In contrast, granularity levels of 5% and 10% may be optimal for displaying smartwatch feedback. We found no significant differences between high-salience and low-salience interfaces in terms of interpretive accuracy and subjective perceptions. Interestingly, participants who had previously used a smartwatch but were no longer using one exhibited larger errors in interpreting progress feedback. However, both previous and current smartwatch users reported higher subjective perceptions compared to those who had never used a smartwatch. Our results extend the research on glanceable feedback and can help researchers design effective glanceable smartwatch feedback displays.

## Data Availability

The datasets analyzed during this study are available from the corresponding author upon reasonable request.

## Appendix

Multimedia Appendix 1 Model diagnostics and secondary tables for the findings.

## Abbreviations

GEE: generalized estimating equation
MTurk: Mechanical Turk
