# Supplementary material for: Exploring the Design Space of Glanceable Smartwatch Feedback Displays: Experimental Study
Source: JMIR Mhealth Uhealth. 2026 Jul 23;14:e81972. doi: 10.2196/81972 (PMC13448195; doi:10.2196/81972)
Supplement: Supplementary file 1 [file mhealth_v14i1e81972_app1.docx]

**Multimedia Appendix 1**

Model diagnostics and supporting tables for the findings.

Fig S1. GEE model diagnosis. We assessed the residual distributions to ensure the validity of our statistical inferences. By cross-referencing the residuals with the original integer error values (Abs Score), we confirmed that the observed striations in the diagnostic plots are an artifact of the discrete nature of the raw data, rather than a sign of model misspecification.

Table S1. Linear regression analysis of the effects of stylization and salience on *noticeability* scores (reference group: people who have never used a smartwatch before).

|  | Coefficient | Standard error | t | *P* | [0.025 | 0.975] |
| --- | --- | --- | --- | --- | --- | --- |
|  |  |  |  |  |  |  |
| **Intercept** |  |  |  |  |  |  |
|  | 2.734 | 0.312 | 8.775 | <.001 | 2.120 | 3.349 |
| **Stylization** |  |  |  |  |  |  |
|  | -0.037 | 0.073 | -0.513 | .61 | -0.181 | 0.106 |
| **Salience** |  |  |  |  |  |  |
|  | 0.051 | 0.071 | 0.716 | .47 | -0.090 | 0.192 |
| **Age** |  |  |  |  |  |  |
|  | 0.009 | 0.007 | 1.225 | .22 | -0.005 | 0.023 |
| **Gender** |  |  |  |  |  |  |
|  | 0.032 | 0.075 | 0.429 | .67 | -0.116 | 0.181 |
| **Current user** |  |  |  |  |  |  |
|  | 0.354 | 0.182 | 1.944 | .053 | -0.005 | 0.712 |
| **Previous user** |  |  |  |  |  |  |
|  | 0.127 | 0.175 | 0.729 | .47 | -0.217 | 0.472 |

Table S2. Linear regression analysis of the effects of granularity on *noticeability* scores (reference group: 1% granularity).

|  | Coefficient | Standard error | t | *P* | [0.025 | 0.975] |
| --- | --- | --- | --- | --- | --- | --- |
|  |  |  |  |  |  |  |
| **Intercept** |  |  |  |  |  |  |
|  | 3.190 | 0.100 | 31.947 | <.001 | 2.993 | 3.387 |
| **Granularity = 4%** |  |  |  |  |  |  |
|  | 0.027 | 0.208 | 0.128 | .90 | -0.383 | 0.437 |
| **Granularity = 5%** |  |  |  |  |  |  |
|  | -0.309 | 0.240 | -1.289 | .20 | -0.782 | 0.164 |
| **Granularity = 10%** |  |  |  |  |  |  |
|  | 0.325 | 0.200 | 1.622 | .11 | -0.070 | 0.720 |
| **Granularity = 20%** |  |  |  |  |  |  |
|  | -0.023 | 0.256 | -0.091 | .93 | -0.528 | 0.481 |

Table S3. Linear regression analysis of the effects of granularity on *relevance* scores (reference group: 1% granularity).

|  | Coefficient | Standard error | t | *P* | [0.025 | 0.975] |
| --- | --- | --- | --- | --- | --- | --- |
|  |  |  |  |  |  |  |
| **Intercept** |  |  |  |  |  |  |
|  | 3.740 | 0.105 | 35.518 | <.001 | 3.532 | 3.948 |
| **Granularity = 4%** |  |  |  |  |  |  |
|  | 0.193 | 0.219 | 0.882 | .38 | -0.239 | 0.626 |
| **Granularity = 5%** |  |  |  |  |  |  |
|  | -0.550 | 0.253 | -2.174 | .031 | -1.048 | -0.051 |
| **Granularity = 10%** |  |  |  |  |  |  |
|  | 0.230 | 0.211 | 1.087 | .28 | -0.187 | 0.647 |
| **Granularity = 20%** |  |  |  |  |  |  |
|  | 0.038 | 0.270 | 0.140 | .89 | -0.494 | 0.569 |

Table S4. Linear regression analysis of the effects of granularity on *attractiveness* scores (reference group: 1% granularity).

|  | Coefficient | Standard error | t | *P* | [0.025 | 0.975] |
| --- | --- | --- | --- | --- | --- | --- |
|  |  |  |  |  |  |  |
| **Intercept** |  |  |  |  |  |  |
|  | 3.470 | 0.117 | 29.728 | <.001 | 3.240 | 3.700 |
| **Granularity = 4%** |  |  |  |  |  |  |
|  | 0.263 | 0.243 | 1.084 | .28 | -0.216 | 0.743 |
| **Granularity = 5%** |  |  |  |  |  |  |
|  | -0.422 | 0.280 | -1.507 | .13 | -0.975 | 0.130 |
| **Granularity = 10%** |  |  |  |  |  |  |
|  | 0.045 | 0.234 | 0.193 | .85 | -0.417 | 0.507 |
| **Granularity = 20%** |  |  |  |  |  |  |
|  | 0.086 | 0.299 | 0.286 | .77 | -0.504 | 0.675 |

Table S5. Linear regression analysis of the effects of granularity on *usability* scores (reference group: 1% granularity).

|  | Coefficient | Standard error | t | *P* | [0.025 | 0.975] |
| --- | --- | --- | --- | --- | --- | --- |
|  |  |  |  |  |  |  |
| **Intercept** |  |  |  |  |  |  |
|  | 3.420 | 0.110 | 31.208 | <.001 | 3.204 | 3.636 |
| **Granularity = 4%** |  |  |  |  |  |  |
|  | 0.169 | 0.228 | 0.740 | .46 | -0.281 | 0.619 |
| **Granularity = 5%** |  |  |  |  |  |  |
|  | -0.531 | 0.263 | -2.019 | .045 | -1.050 | -0.012 |
| **Granularity = 10%** |  |  |  |  |  |  |
|  | 0.317 | 0.220 | 1.443 | .15 | -0.116 | 0.751 |
| **Granularity = 20%** |  |  |  |  |  |  |
|  | -0.031 | 0.281 | -0.111 | .91 | -0.584 | 0.522 |

Table S6. Linear regression analysis of the effects of granularity on *noticeability* scores (reference group: 10% granularity).

|  | Coefficient | Standard error | t | *P* | [0.025 | 0.975] |
| --- | --- | --- | --- | --- | --- | --- |
|  |  |  |  |  |  |  |
| **Intercept** |  |  |  |  |  |  |
|  | 3.515 | 0.174 | 20.222 | <.001 | 3.172 | 3.858 |
| **Granularity = 1%** |  |  |  |  |  |  |
|  | -0.325 | 0.200 | -1.622 | .11 | -0.720 | 0.070 |
| **Granularity = 4%** |  |  |  |  |  |  |
|  | -0.298 | 0.252 | -1.185 | .24 | -0.795 | 0.198 |
| **Granularity = 5%** |  |  |  |  |  |  |
|  | -0.634 | 0.279 | -2.275 | .024 | -1.184 | -0.085 |
| **Granularity = 20%** |  |  |  |  |  |  |
|  | -0.348 | 0.293 | -1.191 | .24 | -0.925 | 0.229 |

Table S7. Linear regression analysis of the effects of granularity on *comprehension* scores (reference group: 10% granularity).

|  | Coefficient | Standard error | t | *P* | [0.025 | 0.975] |
| --- | --- | --- | --- | --- | --- | --- |
|  |  |  |  |  |  |  |
| **Intercept** |  |  |  |  |  |  |
|  | 4.091 | 0.192 | 21.299 | <.001 | 3.712 | 4.470 |
| **Granularity = 1%** |  |  |  |  |  |  |
|  | -0.656 | 0.222 | -2.961 | .003 | -1.093 | -0.219 |
| **Granularity = 4%** |  |  |  |  |  |  |
|  | -0.591 | 0.278 | -2.123 | .035 | -1.140 | -0.042 |
| **Granularity = 5%** |  |  |  |  |  |  |
|  | -1.091 | 0.308 | -3.542 | <.001 | -1.698 | -0.484 |
| **Granularity = 20%** |  |  |  |  |  |  |
|  | -0.702 | 0.323 | -2.171 | .031 | -1.340 | -0.064 |

Table S8. Linear regression analysis of the effects of granularity on *relevance* scores (reference group: 10% granularity).

|  | Coefficient | Standard error | t | *P* | [0.025 | 0.975] |
| --- | --- | --- | --- | --- | --- | --- |
|  |  |  |  |  |  |  |
| **Intercept** |  |  |  |  |  |  |
|  | 3.970 | 0.183 | 21.657 | <.001 | 3.608 | 4.331 |
| **Granularity = 1%** |  |  |  |  |  |  |
|  | -0.230 | 0.211 | -1.087 | .28 | -0.647 | 0.187 |
| **Granularity = 4%** |  |  |  |  |  |  |
|  | -0.036 | 0.266 | -0.137 | .89 | -0.560 | 0.487 |
| **Granularity = 5%** |  |  |  |  |  |  |
|  | -0.779 | 0.294 | -2.651 | .009 | -1.359 | -0.200 |
| **Granularity = 20%** |  |  |  |  |  |  |
|  | -0.192 | 0.309 | -0.622 | .53 | -0.800 | 0.417 |

Table S9. Linear regression analysis of the effects of granularity on *complexity* scores (reference group: 10% granularity).

|  | Coefficient | Standard error | t | *P* | [0.025 | 0.975] |
| --- | --- | --- | --- | --- | --- | --- |
|  |  |  |  |  |  |  |
| **Intercept** |  |  |  |  |  |  |
|  | 3.576 | 0.232 | 15.400 | <.001 | 3.118 | 4.034 |
| **Granularity = 1%** |  |  |  |  |  |  |
|  | -0.766 | 0.268 | -2.860 | .005 | -1.294 | -0.238 |
| **Granularity = 4%** |  |  |  |  |  |  |
|  | -0.542 | 0.336 | -1.612 | .11 | -1.206 | 0.121 |
| **Granularity = 5%** |  |  |  |  |  |  |
|  | -1.195 | 0.372 | -3.209 | .002 | -1.929 | -0.461 |
| **Granularity = 20%** |  |  |  |  |  |  |
|  | -0.659 | 0.391 | -1.686 | .09 | -1.430 | 0.112 |

Table S10. Linear regression analysis of the effects of granularity on *division of attention* scores (reference group: 10% granularity).

|  | Coefficient | Standard error | t | *P* | [0.025 | 0.975] |
| --- | --- | --- | --- | --- | --- | --- |
|  |  |  |  |  |  |  |
| **Intercept** |  |  |  |  |  |  |
|  | 3.818 | 0.204 | 18.719 | <.001 | 3.416 | 4.220 |
| **Granularity = 1%** |  |  |  |  |  |  |
|  | -0.523 | 0.235 | -2.224 | .027 | -0.987 | -0.059 |
| **Granularity = 4%** |  |  |  |  |  |  |
|  | -0.218 | 0.296 | -0.738 | .46 | -0.801 | 0.365 |
| **Granularity = 5%** |  |  |  |  |  |  |
|  | -0.913 | 0.327 | -2.793 | .006 | -1.558 | -0.268 |
| **Granularity = 20%** |  |  |  |  |  |  |
|  | -0.485 | 0.343 | -1.412 | .16 | -1.162 | 0.192 |

Table S11. Linear regression analysis of the effects of granularity on *attractiveness* scores (reference group: 10% granularity).

|  | Coefficient | Standard error | t | *P* | [0.025 | 0.975] |
| --- | --- | --- | --- | --- | --- | --- |
|  |  |  |  |  |  |  |
| **Intercept** |  |  |  |  |  |  |
|  | 3.515 | 0.203 | 17.299 | <.001 | 3.114 | 3.916 |
| **Granularity = 1%** |  |  |  |  |  |  |
|  | -0.045 | 0.234 | -0.193 | .85 | -0.507 | 0.417 |
| **Granularity = 4%** |  |  |  |  |  |  |
|  | 0.218 | 0.294 | 0.741 | .46 | -0.363 | 0.799 |
| **Granularity = 5%** |  |  |  |  |  |  |
|  | -0.468 | 0.326 | -1.435 | .15 | -1.110 | 0.175 |
| **Granularity = 20%** |  |  |  |  |  |  |
|  | 0.040 | 0.342 | 0.118 | .91 | -0.634 | 0.715 |

Table S12. Linear regression analysis of the effects of granularity on *usability* scores (reference group: 10% granularity).

|  | Coefficient | Standard error | t | *P* | [0.025 | 0.975] |
| --- | --- | --- | --- | --- | --- | --- |
|  |  |  |  |  |  |  |
| **Intercept** |  |  |  |  |  |  |
|  | 3.737 | 0.191 | 19.591 | <.001 | 3.361 | 4.114 |
| **Granularity = 1%** |  |  |  |  |  |  |
|  | -0.317 | 0.220 | -1.443 | .15 | -0.751 | 0.116 |
| **Granularity = 4%** |  |  |  |  |  |  |
|  | -0.148 | 0.276 | -0.537 | .59 | -0.694 | 0.397 |
| **Granularity = 5%** |  |  |  |  |  |  |
|  | -0.848 | 0.306 | -2.774 | .006 | -1.452 | -0.245 |
| **Granularity = 20%** |  |  |  |  |  |  |
|  | -0.348 | 0.321 | -1.085 | .28 | -0.982 | 0.285 |
